# Serum thioredoxin is a diagnostic marker for hepatocellular carcinoma

**DOI:** 10.18632/oncotarget.3314

**Published:** 2015-03-19

**Authors:** Jun Li, Zhang-Jun Cheng, Yang Liu, Zhen-Lin Yan, Kui Wang, Dong Wu, Xu-Ying Wan, Yong Xia, Wan Yee Lau, Meng-Chao Wu, Feng Shen

**Affiliations:** ^1^ Department of Hepatic Surgery, The Eastern Hepatobiliary Surgery Hospital, Second Military Medical University, Shanghai, China; ^2^ Department of General Surgery, The Zhongda Hospital, Southeast University, Nanjing, China; ^3^ Department of Chinese Traditional Medicine, The Eastern Hepatobiliary Surgery Hospital, Second Military Medical University, Shanghai, China; ^4^ Faculty of Medicine, The Chinese University of Hong Kong, Shatin, New Territories, Hong Kong

**Keywords:** Thioredoxin, hepatocellular carcinoma, biomarker, diagnosis, Chinese

## Abstract

Here we found that serum levels of thioredoxin were increased in patients with hepatocellular carcinoma (HCC). The optimum diagnostic cutoff for thioredoxin was 20.5 ng/mL (area under curve [AUC] 0.946 [95% CI 0.923–0.969] in the training cohort; 0.941 [0.918–0.963] in the validation cohort). High serum concentrations of thioredoxin differentiated HCC from chronic liver diseases and cirrhosis (0.901 [0.875–0.923] in the training cohort; 0.906 [0.870–0.925] in the validation cohort). Furthermore, a higher proportion of patients with very early HCC had positive results for thioredoxin than for alpha-Fetoprotein (AFP) (73.7% VS.31.6%; *P* < 0.0001). Among AFP-negative patients with very early HCC, 18 (69.2%) of 26 had positive thioredoxin results. Our results indicate that serum thioredoxin complements measurement of AFP in the diagnosis of HCC, especially in very early disease. Combined model (thioredoxin and AFP) showed a significantly greater discriminatory ability as compared with those markers alone.

## INTRODUCTION

Primary liver cancer is the second most common malignancy, and currently results in 360, 000 incident cases and 350, 000 deaths a year in China [1]. In 2008, half of all new cases of liver cancer and related deaths worldwide were estimated to occur in China [2]. Hepatocellular carcinoma (HCC) accounts for 70–80% of all liver cancers. HCC is the second most common cause of cancer associated death in China [3]. Although surgical resection and liver transplantation provided valid approaches to treat HCC, the 5-year recurrence rate after curative resection is still high, up to 54.1–61.5% and ultimately results in poor OS [3]. This dismal outcome is due partly to the lack of an effective method for timely diagnosis, which leads to only 30–40% of patients with HCC being suitable for potentially curative treatments at the time of diagnosis [4]. Effective diagnostic methods to detect HCC at an early stage may result in more effective treatment and extend patient survival. However, the lack of symptoms in the early stage of HCC makes early diagnosis for HCC impractical.

The limitations of ultrasound, the primary radiologic screening modality under current use, include its operator dependence and its poor ability to differentiate malignant from benign nodules in the small cirrhotic liver. Although imaging with triphasic computed tomography scan and magnetic resonance imaging can improve the diagnostic performance, these techniques are time consuming and too expensive for widespread screening at the present time [5]. The search of reliable and efficient biomarkers for a diagnostic and prognostic evaluation of HCC is still an open issue.

Currently, a-Fetoprotein (AFP), des-gamma carboxy-prothrombin (DCP), and tissue polypeptide antigen are the primary biomarkers used in the diagnosis of HCC [6–8]. AFP is the only molecular marker that has been widely used for the diagnosis and detection of HCC. At a cutoff value of 20 ng/mL of serum, AFP shows 60%–80% sensitivity [9]. However, this sensitivity decreases to about 40% for the detection of small tumors [10]. In addition, a significant increase in serum AFP level (20–200 ng/mL) is detected in a considerable number of patients with chronic liver disease [11], including 15%–58% of patients with chronic hepatitis and 11%–47% with cirrhosis [11]. Thus, the identification of a biochemical marker with better sensitivity and/or specificity than AFP could be extremely helpful in improving early diagnosis of HCC.

Impairment or ablation of antioxidant enzymes leading to the accumulation of toxic levels of ROS might initiate or promote a variety of human diseases, e.g., neurodegeneration, cancer development, arthritis, and atherosclerosis [12]. Redox control has emerged as a fundamental biological control mechanism. The thioredoxin system plays a key role in regulating the overall intracellular redox balance. It basically comprises the small redox protein thioredoxin, nicotinamide adenine dinucleotide phosphate, in its reduced form (NADPH), and thioredoxin reductase (ThioredoxinR), a large homodimeric selenzoenzyme controlling the redox state of thioredoxin [13]. Thioredoxin exerts many of its biological activities by reducing a variety of protein thiols, usually having a relatively low molecular weight disulfide. The activity of thioredoxin is regulated by NADPH, which in turn is produced by G6PD, the rate-limiting enzyme of the oxidative HMPS cycle. Two thioredoxins have been cloned: a 54 kDa enzyme (thioredoxin 1) that is found predominantly in the cytoplasm and a 56 kDa enzyme (thioredoxin 2) that contains a mitochondrial import sequence [14].

Elevated serum thioredoxin levels have been observed in many diseases, including diabetes [15], chronic kidney disease [16], severe burn injury [17] and coronary disease [18]. Increased thioredoxin expression has been seen in a variety of malignancies and recent evidence suggests that it may be associated with aggressive tumor growth and poor survival [19–21]. Miyazaki et al. [22] found that elevated serum level of thioredoxin in patients with HCC. Herein, the aims of this study were to determine thioredoxin levels in the serum, and to establish the sensitivity and specificity of serum thioredoxin for diagnosing HCC in Chinese patients with HCC.

## RESULTS

### Thioredoxin and clinical variables

In our study, 25 HCC patients (19 with secondary liver cancer from other primary regions and 6 with history of other solid tumors) in training cohort and 34 (24 with secondary liver cancer from other primary regions and 10 with history of other solid tumors) in validation cohort were excluded. Thus, we recruited 1100 participants overall, 520 in the test cohort (180 with HCC) and 580 in the validation cohort (240 with HCC). In the study patients with HCC, 95(52.8%) were male and median age was 56 years (IQR, 45–64). At diagnosis, 100 (55.6%) patients were classified into Child-Pugh class A; 60 (33.3%) patients into class B; 20 (11.1%) patients into class C. There were 40 (22.2%) patients at TNM stage I, 70 (38.9%) at stage II, 62 (34.4%) at stage III, and 8 (4.4%) at stage IV. The median tumor size was 6.2 (IQR, 2.5–19.5) cm and the number of patients with solitary tumor was 115 (63.9%). Each case of presence of PVT and extra-hepatic metastasis was 55 (30.6%) and 33 (18.3%), respectively. Baseline characteristics of the HCC and control cases were shown in Table 1.

**Table 1:** Baseline characteristics of the HCC and control cases in training cohort

	HCC	LC	CLD	Normal cases
No.	180	120	120	100
Age (IQR, years)	56 (45–64)	56 (44–64)	55 (45–63)	56 (45–64)
Males (%)	52.8	53.3	53.3	53.0
Infection time(IQR, years)	30 (22–39)	28 (19–32)	25 (18–31)^b^	-
Etiology (N)				
HBV	150	95	96	-
HCV	33	27	25	-
Combination(HBV + HCV)	5	3	2	-
Alcohol	2	1	1	-
HBV copies > 10^3^ (N)	134	45^b^	32^b^	-
Tumor size (IQR, cm)	6.2 (2.5–19.5)	-	-	-
Presence of PVT (%)	55 (30.6)	-	-	-
Presence of metastasis (%)	33 (18.3)	-	-	-
Child-Pugh (N)				
Class A	100	78	-	-
Class B	60	32	-	-
Class C	20	10	-	-
Grading(N)				
G1	113	-	-	-
G2/3	67	-	-	-
Tumour stage^a^				
I	40	-	-	-
II	70	-	-	-
III	62	-	-	-
IV	8	-	-	-
No. of nodules(N)				
1	115	-	-	-
> 1	65	-	-	-
Nodule size(N)				
≤ 2 cm	38	-	-	-
> 2 cm	142	-	-	-
Laboratory findings(IQR)				
Platelet counts (×10^3^/mm3)	152 (124–189)	163 (133–199)	165 (132–205)	194 (167–245)^b^
ALT (U/L)	115 (25–282)	136 (35–302)	127 (32–300)	27 (19–32)^b^
AST(U/L)	120 (36–316)	147 (42–330)	155 (50–387)	29 (20–33)^b^
Total bilirubin (mg/dL)	0.92 (0.78–1.42)	1.02 (0.89–1.55)	1.23 (1.01–1.67)	0.55 (0.32–0.87)^b^
Albumin (g/L)	35.2 (32.8–40.4)	36.1 (33.2–41.4)	37.0 (35.2–43.4)	43.2 (39.4–47.6)^b^
Prothrombin time (%)	14.6 (11.2–18.5)	14.2 (10.8–18.2)	13.5 (10.2–18.0)	12.2 (11.5–13.4)^b^
HS-CRP (mg/dl)	0.99 (0.45–1.77)	0.66 (0.35–0.89)^b^	0.59 (0.37–0.85)^b^	0.28 (0.11–0.35)^b^
AFP (ng/ml)	142 (18–548)	15.4 (8.7–30.2)^b^	13.6 (6.8–24.4)^b^	6.6 (4.0–9.2)^b^
TRX (ng/ml)	45.1 (28.2–56.0)	9.0 (6.1–11.9)^b^	8.1 (5.0–10.2)^b^	7.5 (6.0–9.2)^b^

a Tumor was staged accordingly to the American Liver Tumour Study Group modified TNM staging classification.

b *p* value < 0.05, compare with HCC group. HBV, hepatitis B virus; HCV, hepatitis C virus; ALT, alanine aminotransferase; PVT, portal vein thrombosis; AFP, α-fetoprotein; CLD, chronic liver diseases; HCC, Hepatocellular carcinoma; LC, liver cirrhosis; Hs-CRP, High-sensitivity- C-reactive protein; TRX, thioredoxin.

### Main findings

**Figure 1:**
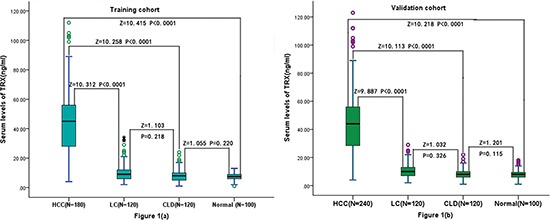
Box plot for serum TRX values in the studied groups **(a)** Box plot for serum TRX values in the training cohort and controls; **(b)** Box plot for serum TRX values in the validation cohort and controls. The box indicates the 25th and 75th percentile of the data, and the middle line, the median. A line extends from the minimum to the maximum value, excluding outliers that are displayed as separate points. CLD = chronic liver diseases; LC = liver cirrhosis; HCC = hepatocellular carcinoma; TRX = thioredoxin.

The median levels of serum thioredoxin was 45.1 (IQR, 28.2–56.0) ng/ml, which was significantly higher than that of healthy subjects, patients with cirrhosis and chronic liver diseases (*P* < 0.0001; Figure 1a). Similarly, thioredoxin was also significantly higher as compared to controls in validation cohort (Figure 1b). Although the median concentration of AFP in serum was increased for patients in the HCC group compared with that in healthy controls, as expected (*P* < 0.0001), significant increases were also seen in patients with LC and CLD (*P* < 0.0001; Table 1). The relationship of thioredoxin with tumor size, Child-Pugh class and tumor stage were evaluated. There was a significant correlation between thioredoxin concentrations and tumor size, Child-Pugh class or tumor stage (*r* = 0.311, *P* < 0.0001; *r* = 0.377, *P* < 0.001; *r* = 0.442, *P* < 0.0001; respectively). The level of thioredoxin tended to increase as liver disease progressed from Child-Pugh class A to C as well as tumor stage from I to IV. For comparison, AFP values of different tumor size or differentiation status or liver function were also analyzed. There was no significantly correlation were obtained. In addition, there was a weak but significant positive correlation between thioredoxin and Hs-CRP (*r* = 0.201, *P* < 0.001). Statistical analysis here revealed no influence of age, sex, infection time, etiology, ALT, AST, total bilirubin, prothrombin time and AFP on thioredoxin in HCC patients (*P* > 0.05, respectively). Similarly results were obtained in the validation cohort. There was also a significant positive correlation between THIOREDOXIN and Hs-CRP (*r* = 0.209, *P* < 0.001).

In univariate logistic regression analysis, thioredoxin as a continuous variable was associated with an increased risk of HCC with an unadjusted OR of 1.39 (95% CI, 1.19–1.72; *P* < 0.001) when controls with LC were included. After adjusting for all other possible covariates, thioredoxin remained can be seen as an independent diagnosis marker of HCC with an adjusted OR of 1.21 (95% CI, 1.13–1.32; *P* < 0.0001). Similarly, thioredoxin as a continuous variable was associated with an increased risk of HCC with an unadjusted OR of 1.45 (95% CI, 1.22–1.91; *P* < 0.001) when controls with LC and CLD were included. After adjusting for all other possible covariates, thioredoxin remained can be seen as an independent diagnosis marker of HCC with an adjusted OR of 1.25 (95% CI, 1.16–1.35; *P* < 0.0001). This relationship was confirmed in the dose-response model (Table 2). When all the HCC patients and controls were included, thioredoxin still was associated with an increased risk of HCC with an unadjusted OR of 1.56 (95% CI, 1.25–1.99; *P* < 0.001). Again, after adjusting for all other possible covariates, thioredoxin remained can be seen as an independent diagnosis marker of HCC with an adjusted OR of 1.33 (95% CI, 1.21–1.54; *P* < 0.001; Table 2.)

**Table 2:** Univariate and Multivariate logistic regression analysis for risk of HCC

Parameter	Univariate Analysis			Multivariate Analysis		
	OR^a^	95% CI^a^	*P*	OR^a^	95% CI^a^	*P*
Training cohort						
Predictor: HCC VS LC and CLD						
Age	1.25	1.08–1.77	0.003	1.20	1.04–1.64	0.004
Sex	1.08	0.87–1.45	0.132	-		
Hs-CRP	1.17	1.08–1.27	0.009	1.11	1.01–1.13	0.012
Family history	13.12	3.12–97.34	< 0.001	10.14	2.11–89.45	0.003
HBV copies > 10^3^	6.32	2.44–18.34	< 0.001	5.55	2.01–15.02	< 0.001
AFP > 20 ng/ml	4.76	2.01–11.32	< 0.001	4.45	1.84–10.18	< 0.001
ALT > 40 U/L	2.11	1.35–3.65	0.009	1.91	1.24–3.19	0.011
Albumin < 4.0 g/dL	1.78	1.09–2.04	0.022	1.45	1.03–2.03	0.231
TRX^a^	1.45	1.22–1.91	< 0.001	1.25	1.16–1.35	< 0.001
TRX(>20.5 ng/mL)	13.22	2.99–87.32	< 0.001	7.03	2.04–15.87	< 0.001
Predictor: HCC VS LC, CLD and normal						
Age	1.28	1.06–1.59	< 0.001	1.24	1.05–1.54	0.001
Sex	1.14	0.98–2.08	0.432	-		
Hs-CRP	1.19	1.08–1.45	< 0.001	1.15	1.04–1.56	0.009
Family history	13.65	3.31–99.87	< 0.001	11.34	1.95–93.87	< 0.001
HBV copies > 10^3^	4.98	2.04–12.43	< 0.001	4.88	1.99–11.78	< 0.001
AFP > 20 ng/ml	5.99	2.04–15.32	< 0.001	5.15	1.77–14.15	< 0.001
ALT > 40 U/L	2.43	1.65–3.65	0.001	2.22	1.41–3.48	0.003
Albumin < 4.0 g/dL	1.98	1.17–2.43	0.015	1.76	1.02–3.01	0.076
TRX^a^	1.56	1.25–1.99	< 0.001	1.33	1.25–1.99	< 0.001
TRX(>20.5 ng/mL)	14.43	2.76–93.21	< 0.001	8.12	1.98–18.32	< 0.001
Validation cohort						
Predictor: HCC VS LC and CLD						
Age	1.24	1.08–1.66	0.001	1.18	1.03–1.50	0.005
Sex	1.12	0.85–1.48	0.176			
Hs-CRP	1.19	1.09–1.26	0.008	1.08	1.02–1.24	0.021
Family history	12.76	3.04–95.32	< 0.001	11.67	1.89–94.23	0.002
HBV copies > 10^3^	6.38	2.40–18.28	< 0.001	5.77	1.89–17.12	< 0.001
AFP > 20 ng/ml	4.54	2.03–11.43	< 0.001	4.34	1.78–10.21	< 0.001
ALT > 40 U/L	2.05	1.32–3.60	0.008	1.87	1.25–3.02	0.018
Albumin < 4.0 g/dL	1.72	1.07–2.11	0.021	1.45	0.98–2.09	0.062
TRX^a^	1.45	1.22–1.91	< 0.001	1.24	1.21–1.54	< 0.001
TRX(>20.5 ng/mL)	13.22	2.99–87.32	< 0.001	7.22	2.09–15.09	< 0.001
Predictor: HCC VS LC, CLD and normal						
Age	1.29	1.06–1.65	< 0.001	1.22	1.11–1.35	< 0.001
Sex	1.17	0.95–2.11	0.409			
Hs-CRP	1.17	1.07–1.36	< 0.001	1.13	1.04–1.53	0.008
Family history	12.09	3.11–94.23	< 0.001	10.99	1.98–89.04	< 0.001
HBV copies > 10^3^	5.05	2.07–12.65	< 0.001	4.75	2.03–10.86	< 0.001
AFP > 20 ng/ml	6.04	2.11–14.98	< 0.001	5.09	1.82–13.89	< 0.001
ALT > 40 U/L	2.56	1.67–3.54	< 0.001	2.18	1.37–3.14	0.003
Albumin < 4.0 g/dL	2.03	1.22–3.19	0.032	1.54	1.06–2.87	0.076
TRX^a^	1.57	1.23–2.02	< 0.001	1.36	1.21–1.86	< 0.001
TRX(>20.5 ng/mL)	15.04	2.06–88.76	< 0.001	8.09	1.93–17.65	< 0.001

a Note that the odds ratio corresponds to a unit increase in the explanatory variable.

OR, odds ratio; CI, confidence interval; HCC, Hepatocellular carcinoma; CLD, chronic liver diseases; LC, liver cirrhosis; Hs-CRP, High-sensitivity- C-reactive protein; HBV, hepatitis B virus; ALT, alanine aminotransferase; AFP, α-fetoprotein; TRX, thioredoxin.

### Thioredoxin has higher sensitivity and specificity than AFP in diagnosis of HCC

**Figure 2:**
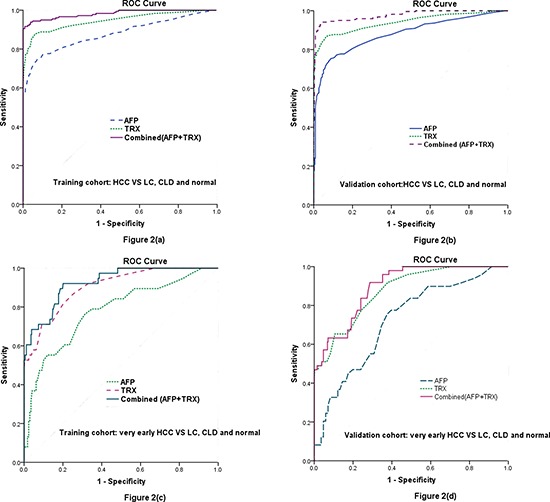
Diagnostic performance of serum TRX and AFP as diagnostic markers for HCC and very early HCC evaluated by the ROC curve **(a)** ROC curve for TRX, AFP, or both for all patients with HCC versus all controls in the training cohort. **(b)** ROC curve for TRX, AFP, or both for all patients with HCC versus all controls in the validation cohort. **(c)** ROC curve for TRX, AFP, or both for patients with very early HCC versus all controls in the training cohort. **(d)** ROC curve for TRX, AFP, or both for patients with very early HCC versus all controls in the validation cohort. ROC = receiver operating characteristics. CLD = chronic liver diseases. LC = liver cirrhosis. HCC = hepatocellular carcinoma; TRX = thioredoxin.

A ROC curve was plotted to define the optimal cut-off values, and to identify the sensitivity and specificity of serum thioredoxin and AFP levels in differentiating patients with HCC versus all other conditions. Based on the ROC curve, the optimal cutoff value of serum thioredoxin levels as an indicator for auxiliary diagnosis of HCC was projected to be 20.5 ng/mL, which yielded a sensitivity of 84.3% and a specificity of 91.8%, with the area under the curve at 0.946 (95% CI, 0.923–0.969); Table 3 and Figure 2a. The optimum cutoff value for AFP was 18.5 ng/mL (AUC 0.878, 95% CI: 0.841–0.914, sensitivity 78.4%, specificity of 81.3%). As the sensitivity and specificity were similar to those for the recommended clinical cutoff of 20 ng/mL (80.1% and 85.9%, respectively; *P* = 0.231), we chose 20 ng/mL as the cutoff value for AFP in this study. Predictive values for thioredoxin and AFP in the diagnosis of HCC are shown in Table 3. Thioredoxin had a better AUROC compared with AFP (*P* < 0.001), indicating both a higher sensitivity and specificity of thioredoxin compared with AFP in the diagnosis of HCC (Figure 2a). When HCC patients were compared with CLD and LC patients, the AUC for thioredoxin was also larger than that for AFP (0.901, 0.875–0.923 *vs.* 0.842, 0.821–0.889, *P* = 0.002; Table 3). Similarly, when HCC patients were compared with LC patients, the AUC for THIOREDOXIN was also larger than that for AFP (0.874, 0.843–0.901 *vs.* 0.824, 0.801–0.853, *P* = 0.004).

**Table 3:** Results for measurement of serum THIOREDOXIN, AFP, or both,* in the diagnosis of HCC

	Training cohort				Validation cohort			
	AUC	95% CI	sensitivity	specificity	AUC	95%	sensitivity	specificity
HCC *vs* LC, CLD and normal								
TRX	0.946	0.923–0.969	84.3%	91.8%	0.941	0.918–0.963	84.1%	91.6%
AFP	0.878	0.841–0.914	78.4%	81.3%	0.880	0.848–0.912	78.6%	81.6%
Combined (TRX + ALP)	0.982	0.970–0.994	88.7%	92.2%	0.978	0.967–0.990	88.7%	92.7%
HCC *vs* LC and CLD								
TRX	0.901	0.875–0.923	78.2%	87.5%	0.906	0.870–0.925	78.7%	87.8%
AFP	0.842	0.821–0.889	74.2%	79.2%	0.840	0.820–0.884	74.0%	79.1%
Combined (TRX + ALP)	0.942	0.922–0.967	86.4%	89.1%	0.941	0.921–0.967	86.6%	89.4%
Very early HCC *vs* LC, CLD and normal								
TRX	0.854	0.825–0.925	74.9%	86.7%	0.901	0.854–0.948	75.2%	88.9%
AFP	0.720	0.646–0.794	68.6%	75.2%	0.769	0.687–0.856	70.1%	79.4%
Combined (TRX + ALP)	0.889	0.866–0.913	81.3%	93.4%	0.917	0.856–0.956	83.1%	94.0%
Very early HCC *vs* LC and CLD								
TRX	0.844	0.812–0.903	74.5%	79.6%	0.847	0.823–0.886	74.8%	80.1%
AFP	0.729	0.684–0.838	70.1%	69.8%	0.727	0.681–0.840	71.2%	77.5%
Combined (TRX + ALP)	0.875	0.842–0.920	81.6%	87.4%	0.870	0.839–0.915	80.5%	86.9%

* The diagnostic cutoff values of serum THIOREDOXIN and AFP were 20.5 ng/mL and 20 ng/mL, respectively.

AUC: area under curve; CI, confidence interval; HCC, Hepatocellular carcinoma; CLD, chronic liver diseases; LC, liver cirrhosis; AFP, α-fetoprotein; TRX, thioredoxin.

Using the optimal cut-offs derived from the ROC curve, thioredoxin was positive in 40 of 55 (72.7%) patients with an AFP < 20 ng/ml. In contrast, AFP was positive in 14 of 34 (41.2%) patients with a thioredoxin < 20.5 ng/mL. Combined model (THIOREDOXIN and AFP) increased the sensitivity for HCC to 88.7%, with a specificity of 92.2%, and the AUROC was 0.98 (95% CI: 0.97– 0.99; Figure 2a). Combined model showed a significantly greater discriminatory ability as compared with those markers alone (Table 3). In the assessment of validation cohort, serum thioredoxin also had greater AUC, sensitivity, and specificity values than did AFP in patients with HCC compared with control cases (Figure 2b and Table 3). Combined model was better than those alone. Based on the ROC curve, the optimal cutoff value of serum thioredoxin levels as an indicator for auxiliary diagnosis of HCC was projected to be 20.9 ng/mL, which yielded a sensitivity of 84.1% and a specificity of 91.6%, with the area under the curve at 0.941 (95% CI, 0.918–0.963). The optimum cutoff value for AFP was 18.4 ng/mL (AUC 0.880, 95% CI: 0.848–0.912, sensitivity 78.6%, specificity of 81.6%). Thus, the diagnostic cut-off values of serum thioredoxin and AFP were also chosen 20.5 ng/mL and 20 ng/mL, respectively.

In the training cohort, 38 (21.1%) of 180 patients with HCC had very early disease. Levels of thioredoxin in serum were significantly higher in these patients than those in all controls [29.2(IQR, 20.1–37.6) vs. 8.2 (5.3–10.6) ng/ml; *P* < 0.0001]. Serum thioredoxin improved differential diagnosis of very early HCC from all controls and from controls at risk of HCC, compared with AFP (Figure 2c, Table 3). When HCC patients were compared with CLD and LC patients, the AUC for thioredoxin was larger than that for AFP (0.854, 0.812–0.903 *vs.* 0.729, 0.684–0.838, *P* < 0.0001; Table 3). Again, when HCC patients were compared with LC patients, the AUC for thioredoxin was also larger than that for AFP (0.801, 0.767–0.845 *vs.* 0.703, 0.665–0.763, *P* = 0.003). Furthermore, a higher proportion of patients with very early HCC had positive results for thioredoxin than for AFP (73.7% VS.31.6%; *P* < 0.0001). Among AFP-negative patients with very early HCC, 18 (69.2%) of 26 had positive thioredoxin results. With use of the 20.5 ng/mL threshold for thioredoxin, we observed similar results in the validation cohort to those in the test cohort. Serum thioredoxin also had greater AUC, sensitivity, and specificity values than did AFP in patients with very early HCC compared with control cases (Figure 2d, Table 3). Combined model was better than those alone.

Interestingly, in training cohort, 132 out of the 180 HCC patients were with AST and/or ALT elevation (>40 U/L). When HCC patients were compared with CLD and LC patients, the AUC for thioredoxin was larger than that for AFP (0.832, 0.799–0.884 *vs.* 0.732, 0.674–0.815, *P* < 0.0001). Again, when HCC patients were compared with LC patients, the AUC for thioredoxin was also larger than that for AFP (0.805, 0.769–0.842 *vs.* 0.704, 0.661–0.763, *P* = 0.002). Furthermore, combined model (thioredoxin and AFP) showed a significantly greater discriminatory ability as compared with those markers alone.

## DISCUSSION

HCC represents an extremely poor prognostic cancer that remains one of the most common and aggressive human malignancies worldwide. The early diagnosis of HCC use of serological markers is of great clinical desirable and the improved prognosis of HCC if the patients could get surgical treatment early [1]. Up to now, AFP has mainly been used in clinic for diagnosis of primary HCC; however, its sensitivity and specificity are not satisfying [23], novel biomarkers for early HCC diagnosis are greatly needed. This study was performed in order to discover valuable non-invasive serum biomarkers which could be obtained without any invasive manipulations such as biopsy for the earliest diagnosis of HCC.

Thioredoxin is involved in various biological activities, including regulating cancer cell growth. These biological activities highlight the importance of redox processes in the pathophysiology of cancer. Park et al. [24] suggested that serum thioredoxin 1 is useful for the early diagnosis of ovarian cancer. We firstly suggested that serum thioredoxin may be a novel marker of HCC in Chinese sample. Similarly, Miyazaki et al. [22] suggesting that measurement of serum of thioredoxin might be a useful clinical parameter when HCC is suspected. However, in our study, the ROC curve comparing all the HCC patients showed that thioredoxin was superior to AFP in diagnosing HCC with an AUROC of 0.95 (95% CI: 0.92–0.97) and a sensitivity of 84.3%, and a specificity of 91.8% (AFP: 0.88). Our findings suggested an advantage of thioredoxin over AFP as a serum marker for detection of HCC, especially for patients with very early HCC and AFP-negative status. The combined measurement of thioredoxin and AFP can further increase the sensitivity for the detection of HCC. In addition, the level of thioredoxin tended to increase as liver disease progressed from Child-Pugh class A to C as well as tumor stage from I to IV. We have found that the elevation of serum THIOREDOXIN was significantly higher in patients with HCC as compare with cirrhosis. Therefore, serum thioredoxin can be used to monitor disease progression in patients with HCC.

Although the diagnostic role of serum AFP in advanced HCC is well recognized, at least one third of small HCCs and up to 40% of advanced HCC will be missed unless other diagnostic tools are used [25]. Similarly, in our study, 30.6% (55/180) of the HCC patients and 68.4% (26/38) of the very-early HCC patients were AFP-negative (<20 ng/mL). In addition, AFP concentrations are raised in 11–58% of patients with chronic hepatitis or cirrhosis in the absence of HCC [26], which was supported by our results. These shortcomings have motivated many investigators to search for other better tumor markers for HCC.

In previous studies, several new biomarkers, such as Golgi protein-73 [27], glypican 3 [9], nidogen-2 [28], heat shock protein 70 [29], and glutamine synthetase [27], ATP-dependent DNA helicase II [3], Des-c-carboxy prothrombin (DCP) [30], squamous cell carcinoma antigen (SCCA) [31], miR-24–3p [32] and miR-483–5p [33] have been investigated for their diagnostic performance and potential clinical application. However, as sensitivity and specificity values of these serological markers were low, they proved to be inadequate for HCC screening purposes, even when combined [34–35]. Few markers have been introduced into the clinic over, mainly because they have not met the following criteria: specific overexpression in cancer cells but not in corresponding normal cells; secreted proteins that can be easily detected in serum; and rare expression in human adult normal tissues except in embryonic tissues [26]. Thioredoxin meets these criteria. Therefore, this protein might have potential as a cancer-specific serum biomarker for various human cancers, including HCC. In this study, we found that thioredoxin was better than AFP for the diagnosis of HCC and very early HCC. In addition, measurement of thioredoxin in serum can help to make a differential diagnosis of HCC in patients in high-risk populations. Further validation studies are needed to confirm the role of thioredoxin in the early detection of HCC.

Whether higher circulating thioredoxin level is an accelerator or only is a marker of HCC remains uncertain. It is important to discuss whether thioredoxin in HCC patients have pathological roles or just was as indicator of oxidative stress or inflammation. Serum thioredoxin levels have been reported to decline significantly after surgical removal of HCC, and may be produced and secreted by HCC cells [36]. When thioredoxin levels are elevated there is increased cell growth and resistance to the normal mechanism of programmed cell death. An increase in thioredoxin levels seen in many human primary cancers compared to normal tissue appears to contribute to increased cancer cell growth and resistance to chemotherapy [37]. Thioredoxin stimulates the growth of a variety of tumor cell lines [38]. Mechanisms by which thioredoxin increases cell growth include an increased supply of reducing equivalents for DNA synthesis [39], reduce hydrogen peroxide [40] and to function as an electron donor to human plasma glutathione peroxidase [41], activation of transcription factors that regulate cell growth [37], and an increase in the sensitivity of cells to other cytokines and growth factors [42]. Because of its role in stimulating cancer cell growth and as an inhibitor of apoptosis, thioredoxin offers a target for the development of drugs to treat and prevent cancer. Importantly, it was previously reported that serum levels of thioredoxin, which is a stress-induced protein, increase relative to the degree of hepatic fibrosis, and that high serum concentrations of thioredoxin may indicate advanced hepatic fibrosis [43]. Correspondingly, Sumida et al. [36] suggested that thioredoxin may therefore be responsible for the pathological mechanism of HCV-related hepatic fibrogenesis, and Tamai et al. [44] reported that serum thioredoxin levels were potential clinical biomarkers that predict patient prognosis in HCV-related HCC. Thus, thioredoxin might play important role in the process of HCC rather than just was a diagnosis marker.

### Strengths and limitations

There have been a few papers in the literature linking thioredoxin and HCC [22, 44], fewer linking very early HCC and AFP-negative status and, as far as I could find, none in an ethnic Chinese sample. As such this manuscript adds significantly to the literature, especially as HCC of ethnic Chinese origin make up such a high proportion of the world and the results are likely to be generalizable to a non-Chinese population. In addition, this study included test and validation cohort which came from different cities, and we were able to control for multiple variables.

Several limitations of this study should be noted. First, in the present study, the classical determination of thioredoxin by ELISA method is widely used, which is considered ‘gold standard’. However, it is widely known that human serum contains immunoglobulins that affect the results of immunoassays by binding to reagent antibodies used in the assay. HAb interference can frequently cause a false positive signal [45]. Second, without serial measurement of the circulating thioredoxin levels, this study yielded no data regarding when and how the change of serum levels in these patients. In addition, thioredoxin measurements were performed after the HCC and may not accurately reflect pre-HCC exposure. Third, we measured only thioredoxin, which is a single antioxidant defense parameter. Effective antioxidant protection is provided by the cooperative and sequential actions of several antioxidant enzymes, and non-enzyme antioxidant molecules. So we could not determine the association of those factors with thioredoxin levels and HCC. Fourth, we measured thioredoxin in serum, not in histologic specimen. It is still uncertain whether peripheral thioredoxin levels reflect similar changes in the liver tissue. However, we did not get relevant information in this study. The relationship between peripheral and tissue thioredoxin levels warrants further investigation. Lastly limitation is the lack of outcome data. This study yielded no data regarding whether treatment can change serum levels in these patients. In addition, we did not obtain the information about antiviral therapy against HBV and/or HCV. So we could not determine the association of antiviral therapy with thioredoxin levels and HCC. Interestingly, Sumida et al. [36] indicated that the mean thioredoxin value was lower in interferon (IFN) responders than in non-responder in patients with type c chronic hepatitis. However, sequence change in serum thioredoxin during IFN therapy and the correlation between thioredoxin and the long-term efficacy of INF treatment should be examined in the future.

In conclusion, to the best of our knowledge, this is the first study to report the clinically diagnostic relevance of thioredoxin as a serum protein marker for HCC in a training cohort and an independent validation cohort. Our results indicated that serum thioredoxin could complement measurement of AFP in the diagnosis of HCC, especially very early disease, and will help to resolve the deficiencies of AFP in the testing of AFP-negative patients.

## PATIENTS AND METHODS

In our study, training cohort recruited 205 consecutive patients with HCC and 340 controls (120 patients with liver cirrhosis, 120 patients with chronic liver diseases, and 100 healthy individuals) from the Eastern Hepatobiliary Surgery Hospital, Second Military Medical University, Shanghai, China, from December 2010, to December 2013. Validation cohort recruited 274 consecutive patients with HCC and 340 controls (120 patients with liver cirrhosis, 120 patients with chronic liver diseases, and 100 healthy individuals) from the Zhongda Hospital, Southeast University, Nanjing, China between May 2011 and March 2014. All patients with first-ever HCC underwent curative resection.

Subjects with other conditions, which may alter thioredoxin, such as previous/concomitant other neoplasm, inflammatory, diabetes, chronic kidney disease, severe burn injury and cardiovascular diseases, were excluded. The healthy subjects with normal liver biochemistry, no history of liver disease or alcohol abuse, and no viral hepatitis were enrolled from the Health Physical Examination Center of those two hospitals. There were no significant differences in demographic or clinic pathological characters of patients with HCC between the training and validation cohorts. This study was approved by the Institutional Review Board of the Eastern Hepatobiliary Surgery Hospital of Second Military Medical University and Zhongda Hospital of Southeast University. Informed consent was obtained from each inductee in accordance with the Ethics Committees Guidelines for our institution.

The diagnosis of HCC was made based on guidelines from the Chinese Society of Hepatology and Chinese Society of Infectious Diseases, Chinese Medical Association [46]. The diagnosis of HCC was made either by histopathology or, if not available, by two imaging tests (ultrasound, CT, MRI or angiography) showing an arterial enhancing lesion with a HBV infection background in the Chinese population. The absence of HCC in patients recruited in the HCC-free chronic liver disease groups was confirmed by ultrasonography at recruitment and 6 months thereafter. HBV infection status was based on hepatitis B surface antigen (HBsAg). HCV infection status was based on serum HCV antibody and HCV RNA determination. Cirrhosis was diagnosed by liver biopsy, abdominal sonography (portal systemic shunts, splenomegaly, spotty coarse parenchyma, nodular surface, and round edge), and biochemical evidence of parenchymal damage plus endoscopic esophageal or gastric varices [47]. Patients with cirrhosis who had raised AFP concentrations were required to have undergone imaging by multiple methods (ultrasonography, CT, or MRI) and to have had no evidence of a hepatic mass for at least 3 months before enrolment. A liver biopsy may be obtained to confirm the diagnosis.

Clinical data collected included age, sex, Child–Pugh score, serum alpha-fetoprotein (AFP), viral status, infection time, tumor size and radiological extent of disease. In addition, treatment history, tumor node metastasis (TNM) staging, Edmondson and Steiner grade and etiological risk factors were collated. The tumor size was the largest diameter measured by imaging. HCC and cirrhotic patients were classified into the three (A/B/C) Child–Pugh's grades based on their clinical state [48]. HCC grading was based on the available material according to Edmondson and Steiner [49], and cases were divided into two groups: well-differentiated histology (G1) and moderately to poorly differentiated histology (G2/G3). Tumor staging (I, II, III, IV) was established using the American Liver Tumor Study Group modified TNM staging classification [50]. In our study, very early HCC was defined as well-differentiated, < 2-cm HCC [51].

Blood specimens were drawn prior to initiation of treatment (curative resection) for HCC. Venous blood samples were taken in the morning's fasting state. After at least 30 min, but within 2 h, the tubes were centrifuged at 20°C for 15 min at 1, 200 g, and the sera were stored frozen in plastic vials at-80° until the time of consecutive analyses. The controls samples were collected and stored in the same way as the HCC samples. AFP levels were measured with commercially available immunoassay methods (Axysm). A cut-off value of 20 ng/mL was used. AFP levels greater than or equal to 20 ng/mL were defined as positive. Serum levels of thioredoxin was measured in duplicate using a solid-phase sandwich ELISA that uses two highly specific antibodies to human thioredoxin protein; one antibody is precoated onto the thioredoxin ELISA plate and the other antibody is HRP-conjugated (Immuno-Biological Laboratories Co., Ltd, Gunma, Japan) according to the manufacturer's instruction. The coefficients of variation (CVs) of inter-assay and intra-assay were 6.0–9.1% and 7.2–10.0%, respectively. The lower detection limit was 0.45 ng/mL and the line range was 0.45–100 ng/ml. Other biomarkers, such as white blood cell count (WBC) and high sensitivity C-reactive protein (Hs-CRP) were also tested by standard laboratory method. For all measurements, levels that were not detectable were considered to have *a* value equal to the lower limit of detection of the assay.

Results are expressed as percentages for categorical variables and as medians (interquartile ranges, IQRs) for the continuous variables. Proportions were compared using the Fisher's exact test, and the Mann–Whitney test to compare continuous variables between groups. Correlations among parameters were analyzed by using Spearman's rank correlation test. The influence of serum thioredoxin levels on HCC was performed by binary logistic regression analysis, which allows adjustment for other confounding factors, such as, sex, age, etiology, family history, HbsAg, HBV copies and serum levels of ALT, AST, total bilirubin, prothrombin time and AFP. Results were expressed as adjusted OR (odds ratios) with the corresponding 95% Confidence interval (CI). Receiver operating characteristic (ROC) curves were utilized to evaluate the accuracy of serum biomarkers to diagnose HCC. Area under the curve (AUC) was calculated as measurements of the accuracy of the test. All statistical analysis was performed with SPSS for Windows, version 19.0 (SPSS Inc., Chicago, IL, USA) and STATA 9.2 (Stata Corp, College Station, TX), R version 2.8.1. Two-tailed significance values were used and significance levels were set at 0.05.
